# SUPERA Stenting in the Common Femoral Artery: Early Experience and Practical Considerations

**DOI:** 10.1177/15385744211068648

**Published:** 2022-02-11

**Authors:** Mary Jiayi Tao, Akshat Gotra, Kong Teng Tan, Naomi Eisenberg, Graham Roche-Nagle, Sebastian Mafeld

**Affiliations:** 1 Division of Vascular and Interventional Radiology, Department of Medical Imaging, Joint Department of Medical Imaging, 7938 University of Toronto, Toronto, ON, Canada; 2 Division of Vascular and Interventional Radiology, Department of Medical Imaging, The Ottawa Hospital, 6363 University of Ottawa, Ottawa, ON, Canada; 3 Department of Vascular Surgery, 33540 Toronto General Hospital, Toronto, ON, Canada

**Keywords:** common femoral artery, stents, endovascular therapy, revascularization, self-expanding stent

## Abstract

**Purpose:**

Endovascular therapy in the management of de novo common femoral disease remains controversial. Considerable interest has been generated in recent years due to recent technological advancement in the design of vascular stents. In particular, SUPERA (Abbot Vascular Inc, Santa Clara USA) stents are designed to offer increased flexibility and less adverse interactions with the arterial wall, thus making it potentially better suited for common femoral lesions. However, despite such theoretical advantages, there is lack of data in its use in clinical practice. This study provides illustrative examples of SUPERA stents in different clinical settings and contributes to important clinical data for the overall efficacy and safety profile of endovascular interventions in common femoral artery (CFA) disease.

**Materials and Methods:**

Retrospective analysis of all endovascular CFA procedures between January 1, 2011, and December 31, 2019, was conducted. Data collected included demographics, clinical symptoms, medical comorbidities, procedural characteristics, and immediate and short-term complications. Detailed analysis was performed on the stenting cohort.

**Results:**

During our study period, a total of 69 patients underwent endovascular interventions involving the CFA at our institution, of which 16 patients had stenting procedures for a total of 18 stent deployments. Technical success was achieved in all stenting procedures. A total of 15 SUPERA stents were placed in 13 patients. No stent fractures were observed. Overall primary patency rate of SUPERA stents at the time of 12-month follow-up was 100% in patients who had a follow-up assessment (n = 12 stents).

**Conclusion:**

Endovascular intervention of the CFA is an evolving topic in the interventional radiology and vascular surgery community. Recent development of newer generation of devices such as SUPERA peripheral stents offers significant potential benefits given their inherent design. Despite the theoretically promising design of the SUPERA, there is a lack of data to support its use. This study contributes important patient-level data for SUPERA stent deployments.

## Introduction

The role of endovascular therapy for common femoral artery (CFA) atherosclerotic disease has been much debated, with femoral endarterectomy currently considered the gold standard with primary patency rates approaching 90% at 5 years.^1,2^ An endovascular first approach for the CFA has not been widely adopted due to its unique anatomic environment involving flexion and torquing forces.^3^ Potential fears include stent kinking, fracture, or development of neointimal hyperplasia leading to occlusion.^4^ Furthermore, CFA stent placement risks jailing the profunda femoris artery, a major collateral vessel for the leg. Hypothetically, stenting may also preclude future endovascular or surgical access to the CFA.

In recent years, evidence for endovascular CFA treatment has evolved, showing high rates of technical success with emerging data for primary/secondary patency and freedom from target lesion/extremity revascularization. Technological developments such as vascular mimetic stents (SUPERA Abbot Vascular Inc, Santa Clara, USA) may be better suited to deal with the eccentric calcified plaques and crush risk attributed to the CFA.^5^ In addition, these stents may preserve future endovascular accessibility of the CFA.^5^ Recently, data from a randomized controlled trial (TECCO Trial) comparing the safety and efficacy of stenting vs open surgery for de novo CFA stenosis^6^ found that perioperative morbidity and mortality was lower among patients who underwent endovascular therapy with stenting compared to surgery.^6^ Consequently, endovascular treatment of the CFA is gaining more consideration as an alternative treatment to surgery or in patients who are not surgical candidates. Given the availability of a large vascular database at our center which collects data on each endovascular procedure, this study examines our experience with CFA endovascular stenting with focus on SUPERA stents with regard to safety and clinical efficacy over a 9-year period.

## Materials and Methods

Institutional Research Ethics Board approval (UHN Research CAPCR Study ID #19-6174.0) was obtained to perform a retrospective review of all patients who had an endovascular CFA intervention between January 1, 2011, and December 31, 2019. Requirement for informed consent was waived. Eligible patients were identified using the centralized Vascular Quality Initiative database, a clinical registry which prospectively collects robust data from patients treated by vascular specialists in academic and community hospitals across North America. Patients were included if intervention was performed for CFA stenosis or occlusive disease. Exclusion criteria included interventions performed for non-atherosclerotic disease including iatrogenic dissection or bleeding.

### Data Collection

Each eligible patient was reviewed for age; gender; presenting symptoms; lesion type; pre-procedural duplex and anklebrachial indexes; and intervention characteristics including access, type of stenting, technical success, and complications. Perioperative morbidity and mortality within 30 days of the procedure included death from any cause.

**Figure 1.**
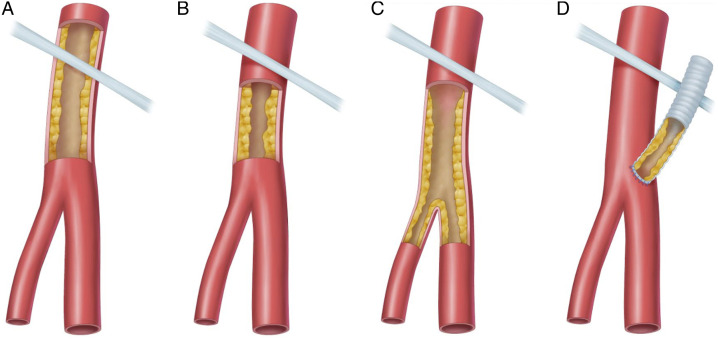
Classification of common femoral artery disease. A: Type I lesions; B: Type II lesions; C: Type III lesions; D: Type IV lesions

Common femoral artery lesions were classified into 4 categories (Figure 1). Type I lesions were located at the iliac external artery and extended into the CFA, type II lesions were limited to the CFA, type III lesions involve the CFA and femoral bifurcation, and type IV lesions represented anastomotic stenosis involving the CFA.

Follow-up information included post-procedural duplex and anklebrachial indexes and symptoms. Patients were deemed lost to follow-up when no further follow-up visits or evaluations were available at our institution at time of data extraction.

### Procedural Technique

All patients were seen and assessed by a staff vascular surgeon and/or interventional radiologist prior to the procedure. All patients included in this study were declined for surgery and referred to endovascular treatment as it is local practice to perform endarterectomy for significant common femoral artery stenoses. Local anesthesia with conscious sedation was provided. Arterial access into the common femoral artery or brachial artery was achieved using ultrasound guidance. Following vascular sheath placement, a digital subtraction angiogram was performed to visualize the CFA and the bifurcation. Various catheters and guidewires were used to navigate to the target lesion. Pre-stenting angioplasties were performed in all patients, and stents were inserted when the residual stenosis was more than 30% of the native artery diameter following angioplasty. In such cases, patients received SUPERA stents (Abbott Vascular, Chicago, IL) or self-expandable stents (ZILVER, Cook, Bloomington, IN; EPIC and INNOVA, Boston Scientific, Boston, MA). Stent sizing selection was based on the vessel diameter, and was equal to or 1 mm less than the pre-stenting angioplasty balloon size. Post-stent deployment angiograms were performed to ensure satisfactory post-stenting results.

### Technical Success and Clinical Outcomes

Procedural success was defined as the ability to traverse the lesion and deploy a stent. The primary endpoint of primary patency at 12 months was defined as lack of restenosis or need for re-intervention of the symptomatic target CFA lesion. Follow-up information included clinical and hemodynamical assessments based on clinical symptoms, duplex evaluation, and anklebrachial index measurements as appropriate. CT angiograms were performed in cases of worsening clinical symptoms and/or findings suggestive of hemodynamic changes on duplex scans.

## Results

**Figure 2.**
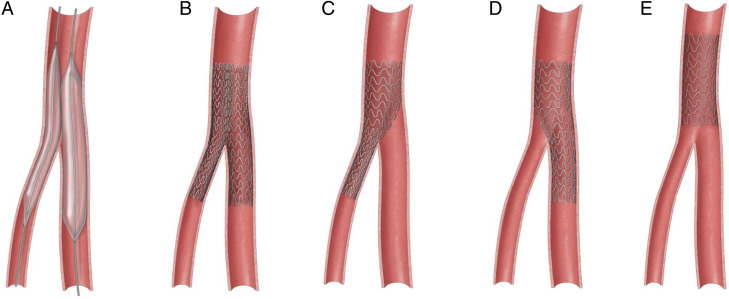
Various endovascular techniques for femoral bifurcation lesions. A: Kissing balloon; B: Kissing stents; C: CFA to profunda jailing the SFA; D: CFA to SFA jailing the profunda; E: Isolated CFA stent

During our study period, a total of 69 patients underwent endovascular interventions involving the common femoral artery at our institution. Of the 69 endovascular procedures performed at our institution, endovascular stent placements were performed in 16 patients with a total of 18 stent deployments. Individual cases including patient demographics, presenting symptoms, CFA vessel characteristics, stent type, pre- and post-procedural duplex, complications, and outcomes are outlined in Table 1. Average age of this subgroup is 70.2 years old, and 56.2% were male. The most common access site is the femoral artery (n = 13) with the up-and-above technique utilized in most patients (n = 11). Type III CFA lesions were the most intervened (n = 6) in this subgroup. SUPERA stents (Abbott Vascular, Chicago, IL) were placed in all patients with de novo CFA lesions, while self-expandable stents (ZILVER, Cook, Bloomington, IN; EPIC and INNOVA, Boston Scientific, Boston, MA) were inserted into anastomotic stenotic segments of the CFA. Each pre-deployment and post-deployment angiogram was closely examined, and the location of stent placement was determined based on Figure 2. Single isolated CFA stents were placed in 8 patients; single jailed profunda and SFA stents were placed in 4 and 2 patients, respectively, while 2 patients had double kissing stents deployed into the superficial femoral artery (SFA) and profunda. Technical success was achieved in all cases.

**Table 1.** CFA Stenting Outcomes.

Patients	Age	Gender	Symptoms	Access Site	TASC	CFA	Pre-Procedure	Stent	Stent Placement	Complications	30-day	Primary Patency	Post-Intervention	Post-Procedure
					Score	Type	Duplex PSVR	(type, diameter x length in mm)			Mortality	(12-months)	Symptoms	Duplex PSVR
**1**	57	M	Tissue loss	Femoral retrograde	2	2	0.50	ZILVER 8 x 40	Single, isolated CFA	N	N	Y	Asymptomatic	0.79
**2**	83	M	Tissue loss	Femoral retrograde	1	3	0.42	SUPERA (7 x 40)	Single, isolated CFA	Osteomyelitis	N	Y	Asymptomatic	0.69
**3**	62	M	Acute ischemia	Femoral retrograde	2	4	27.9	EPIC (8 x 40)	Single, jailed SFA	N	N	Y	Asymptomatic	1.63
**4**	73	M	Tissue loss	Femoral retrograde	1	3	2.48	SUPERA (7 x 40)	Single, isolated CFA	N	N	Y	Improved	0.53
**5**	48	F	Tissue loss	Brachial	2	2	0.85	SUPERA (5 x 80)	Single, jailed SFA	N	Y	N/A	N/A	N/A
**6**	61	M	Claudication (short distance)	Femoral retrograde	3	1	1.57	SUPERA (7 x 40)	Single, isolated CFA	N	N	Y	Worsen	2.44
**7**	91	F	Ischemic rest pain	Femoral retrograde	2	2	0.71	SUPERA (7 x 40)	Single, jailed profunda	N	N	Y	Asymptomatic	0.58
**8**	93	F	Acute ischemia	Femoral retrograde	1	3	2.24	SUPERA (4 x 40, 4 x 40)	Double, kissing	NSTEMI	N	Y	Improved	1
**9**	59	F	Acute ischemia tissue loss	Femoral retrograde	1	4	1.26	SUPERA (6x40)	Single, jailed profunda	N	N	Y	Worsen	None
**10**	91	M	Ischemic rest pain	Femoral retrograde	1	2	5.49	SUPERA (7 x 40, 7 x 40)	Double, kissing	N	N	Y	Improved	None
**11**	70	M	Ischemic rest pain	Femoral retrograde	3	1	3.42	SUPERA (6 x 40)	Single, isolated CFA	N	N	N/A*†*	N/A*†*	N/A*†*
**12**	65	M	Ischemic rest pain	Brachial	1	4	N/A	SUPERA (7 x 60)	Single, isolated CFA	N	N	Y	Asymptomatic	1.46
**13**	66	M	Acute ischemia	Femoral retrograde	2	1	N/A	SUPERA (5 x 80)	Single, jailed profunda	N	N	Y	No change	Patent^a^
**14**	71	F	Tissue loss	Femoral retrograde	1	3	1.03	SUPERA (6 x 40)	Single, isolated CFA	GI Bleed	N	Y	Improved	1.25
**15**	82	F	Ischemic rest pain	Femoral retrograde	1	3	N/A	SUPERA (5 x 200)	Single, jailed profunda	No follow-up	No follow-	No follow-up	No follow-up	No follow-up
**16**	52	F	Ischemic rest pain	Brachial	1	3	0.72	INNOVA (7 x 150)	Single, isolated CFA		up	Y	Worsen	0.90

Abbreviations: CFA, common femoral artery; PSVR, peak systolic velocity ratio; SFA, superficial femoral artery.

^a^Velocity not obtained † Patient 11 passed away within 12 months of the intervention due to nature causes.

One patient died within 30 days of the intervention to a cause deemed unrelated to the intervention, while another patient died within 12 months of the procedure due to natural causes. Major complications in 30 days following endovascular stenting were NSTEMI (n = 1), osteomyelitis (n= 1), and gastrointestinal bleeding (n = 1). Baseline duplex stenotic peak velocity ratio decreased from 3.74 ± 7.11 to 1.12 ± 0.56 (P = .11) following intervention. Overall improvements in symptoms were documented in 9 patients, while 1 patient did not notice any changes in overall clinical symptoms. Three patients experienced worsening symptoms within 12 months, with documented elevated peak systolic velocity compared to baseline duplex that required further nonCFA-related interventions. Further analysis of each of these 3 patients revealed preserved patency of the CFA stents but had interventions relating to stenoses involving non-CFA lesions. At the time of 12-month follow-up, all patients who returned for reassessment had preserved patency of the in-situ endovascular stent with a primary patency of 100% (n = 15 stents). Specifically, the overall primary patency rate of the SUPERA stent at the time of 12-month follow-up was 100% in our cohort (n = 12 stents).

## Discussion

Peripheral artery disease is a major cause of morbidity and mortality worldwide. In recent years, advancements have been made in the therapeutic options for management of patients requiring lower extremity revascularization. Traditionally, open surgical approaches were considered the gold standard; however, the advent of new technologies and techniques has led to the rapid growth and widespread use of endovascular techniques for treatment of infrainguinal disease. The role of endovascular therapy in the management of de novo common femoral disease remains controversial.

Common femoral artery lesions are challenging and differ from other infrainguinal segments due to their unique anatomical and mechanical properties. During flexion, parts of the legs may be subjected to multiaxial deformations of up to 60% rotation and 20% contraction.^7^ Given the significant morphological changes of shortening, twisting, and bending during the dynamic flexion at the hip, the common femoral artery has long been viewed as a hostile environment for endovascular revascularization given its inherent risk of stent fractures, occlusions, and distal embolization. Beside the primary concerns of perceived inferiority in patency rates compared to open surgical managements, stent placements may limit future surgical or endovascular treatment or access options and potentially compromise the profunda femoral artery. However, in recent years, there has been an emergence of studies examining the overall technical feasibility and clinical outcomes of endovascular interventions of the common femoral artery. This shift in momentum toward minimally invasive techniques is in part because open surgical endarterectomy is not as benign as previously believed and carries complication rates as high as 15% including a 3.4% death rate.^8^

The TECCO trial published in 2017 was the first multicenter randomized controlled trial comparing surgery to stainless-steel stenting for CFA de novo atherosclerotic lesions.^6^ In this study, a total of 117 patients with de novo CFA disease were randomized to the stenting group (n = 56) and endarterectomy group (n = 61). The primary outcome of morbidity and mortality rate within 30 days occurred in 26% of patients in the surgery group and 12.5% in the endovascular group. In particular, there was a higher percentage of local complications including delayed healing (16.4%), paresthesia (6.5%), hematoma (5%), and local infection (5%) in the surgical group. At 24 months, there was no demonstrated difference in the overall sustained clinical improvement, primary patency rate, target lesion, and extremity revascularization rates between the 2 groups.

In our study, analysis was performed to examine outcomes of endovascular stent implantation in the common femoral artery. Traditionally, stent placements were reserved for suboptimal angioplasties or in the setting of angioplasty-related complications. In recent years, there is growing evidence to suggest that percutaneous stents may be a valid option for CFA stenoses (Table 2).^4-6,9-22^ A major propeller for this momentum change is the invention of newer generations of stents which aim to address the unique challenges of the dynamic forces and atherosclerotic issues of the common femoral artery. Osteoid metaplasia is the formation of lamellar bone-like arterial calcification, characterized by formation of extracellular osteoid matrix composed of osteoblast- and osteoclast-like cells, regulatory osseous cytokines, macrophages, and pericytes.^23^ Due to the high prevalence of osteoid metaplasia in the common femoral artery, especially compared to other vessels such as the common carotid artery,^24^ stents must be able to sustain high radial forces while maintaining a high degree of flexibility. In particular, the SUPERA peripheral stent (Abbott Vascular, Sanata Clara, CA, USA), a helical interwoven Nitinol self-expandable device, has been of great interest recently. In a study conducted by Maleckis et al.^25^ comparing different Nitinol stents, the SUPERA device was shown to have the highest torsional stiffness (959.2 µN·m/°) and radial compressive response while maintaining relatively low axial stiffness (94 N/m) which allows for increased flexibility and potentially less adverse interactions with the arterial wall. Such stent design characteristics theoretically make these devices more attractive as the device of choice given its ability to withstand the flexion forces in this vessel segment. To our knowledge, there is one ongoing study with interim data published on the use of SUPERA stents for de novo common femoral lesions. In this study, the cumulative primary patency rate and cumulative freedom from target lesion revascularization rate was 100% at the 6-month follow-up.^5^ Table 2 summarizes stent data for the CFA to date.

**Table 2.** Summary of Common Femoral Artery Stenting Studies.

Study	Year	Study Period	Study Design	Sample Size	Stent Type	Outcomes	Complications
Deloose et al.	2018	2016ongoing	Prospective multicenter	n = 100	Self-expandable Nitinol - *SUPERA® (Abbott Vascular)*	100% cumulative PP and freedom from TLR at 6 months, up to 210 days	None
Stricker et al.	2020	19952015	Retrospective	n = 7994 limbs	Self-expandable stents	Mean ABI from 0.71 ± 0.17 to 1.03 ± 0.2 after 1 year (p < .001)	23 limb restenosis
					- *Absolute Pro Vascular® (Abbott Vascular)*	Cumulative PP after 1, 3, 5, and 8 years was 96, 90, 78, and 63%, respectively	2 puncture site hematomas, 1 arteriovenous fistula (1), 1 cholesterol embolism, and 1 dissection of the access site artery
					- *Zilver Flex Vascular® (Cook Europe) Wallstent® (Boston Scientific) Sentinol® (Boston Scientific) Epic® (Boston Scientific) Jostent® (Jomed GmbH) Everflex® (Medtronic)*		
					- *Xceed® (Abbott Vascular)*		
Guoëffic et al.	2017	20112013	Randomized control trial	n = 56 stenting	Self-expandable Nitinol (Type 1, 2, and type 3 lesions with SFA occlusion)^a^	Morbidity/mortality 26% in the surgery group vs. 12.5% in the stenting group (OR 2.5, *P* = 0.5)	1 stent fracture
				n = 61 surgery	Balloon-expandable (Type 3 lesions affecting the CFA bifurcation)^a^	Sustained clinical improvement, PP, TLR, and TER at 24 months similar in both groups	3 patients in the stent group converted to surgery
Siracuse et al.	2016	20102015	Retrospective	n = 1014 253 CFA stents	^a^	No difference in patency between PTA, PTA+stent, or stent alone at 1 year	Peri-procedural complications: access site hematoma (5.2%), arterial dissection (2.9%), distal embolization (0.7%), access site stenosis/occlusion (0.5%), and arterial perforation (0.6%)
				23 CFA stent-grafts		23 CFA stent-grafts	
Mehta et al.	2016	20062013	Prospective multicenter	n = 16715 CFA stents for failed atherectomy ± PTA	Self-expanding bare metal^a^	The CFA stent group 100% PP vs. CFA non-stent groups 77% PP (p = 0.0424), follow-up = 42.5 months	1 case of distal embolization requiring bypass in the stenting group
Nasr et al.	2016	20062008	Prospective cohort	N = 3640 limbs	Self-expandable Nitinol *Everflex® (Medtronic)*	Primary sustained clinical improvement 77% (3 years) and 73% (5 years)	In-stent restenosis rate 28%—1 stent fracture
					- *Luminexx® (Bard)*	Freedom from TLR 79% and TER 73%	
					- *Flexstar® (Bard)*		
					Balloon-expandable for Type 3 lesions—*Amiia® (Cordis)*		
Thiney et al.	2015	20092013	Prospective	n = 53	Self-expandable Nitinol (33) or balloon-expandable (23)^a^	Absence of binary restenosis (> 50% re-obstruction of the CFA) 92.5% at 24 months	Stent fracture rate 9% at 1 year (n = 4) - 2 cases of restenosis - 2 cases of thrombosis
				50 limbs primary stenting		- Freedom from TLR 97%, TER 11%	
Linni et al.	2014	20112013	Randomized control trial	n = 40 stenting	Bioabsorbable stent implantation (BASI): balloon-expandable stent made from poly-L-lactide acid (*Remedy® Kyoto Medical*)	1-year PP 80% BASI and 100% CFE (*P* = .007)	6 reconstruction failures in BASI vs. 0 in CFE (*P* = 0.02), 5/6 due to stent occlusion
				n = 40 surgery		1-year SP 84% BASI and 100% CFE (*P* = .01)	
						Repeat TER rates, 2.5% BASI, and 7.5% CFE (*P* = .61)	
						Limb salvage equivalent	
de Blic et al.	2013	20082011	Retrospective	n = 3523 CFA stents	Balloon-expandable stents (11) and self-expandable stents (12)^a^	Technical success 100%	No stent-specific complications specified
						No restenosis in stent	
Soga et al.	2013	20012010	Retrospective	n = 183 111 CFA lesions	Self-expandable - *S.M.A.R.T.® (Cordis)* - *Wallstent® (Boston Scientific)* - Balloon-expandable - *2 Express LD® Boston Scientific*	No significant difference in CFA PP between angioplasty vs. stent placement	1 stent deformity, 0 stent fracture in CFA
				10 CFA stents		- CFA SP lower in the stent group vs. angioplasty (*P* = .02)	
Bonvini et al.	2013	19962007	Retrospective	n = 360	Self-expandable^a^	Failures (> 30% residual stenosis) in 26 cases (7.2%)	Major complications’ rate (requiring surgery): 1.4%
				144 CFA stents		- TLR in 64 of 322 (19.9%) - Use of stents an independent protective factor against procedural failure (OR: 0.20), 1-year restenosis (OR: 0.53) and TLR (OR: 0.49)	Minor complications’ rate (treated percutaneously or conservatively): 5.0%
							Restenosis (> 50%) in 74 patients (27.6%)
Ahn et al.	2012	20092011	Retrospective	n = 61	Self-expandable^a^	Cumulative PP 82% at 6 months, 67% at 12 and 24 months	Thirteen cases considered failures
						- Assisted PP 100% at 21 months	
						- Twenty stents subsequently punctured for future endovascular access	
Calligaro et al.	2011	20052010	Retrospective	n = 17	Polytetrafluoroethylene-covered Nitinol stents *Viabahn® Gore*	2-year PP rate 93.8%	DFA deliberately sacrificed in 1 case (emergent bleeding from CFA) - 2 cases required additional procedure for inflow/outflow stenosis
						- Assisted PP rate 100%	
Azema et al.	2011	20062008	Prospective cohort	n = 36	Self-expandable Nitinol *Everflex® (Medtronic)* - *Luminexx® (Bard)* - *Flexstar® (Bard)* -- Balloon-expandable for Type 3 lesions - *Amiia® (Cordis)*	1 year 1°/2° sustained clinical improvement: 80%, 90% - TLR-free survival: 85%	20% in-stent restenosis - 1 stent fracture
						- TER free survival: 80%	
Paris et al.	2011	19942009	Retrospective	n = 26	Self-expandable^a^	Maintained clinical success 100% (16/16) of claudication patients and 70% (7/10) of CLI at 12 and 31 months - ABI from 0.47 to 0.77 (*P* < 0.001)	In-stent restenosis during first year in two CLI patients (placement of additional stent)
Baumann et al.	2011	19952009	Retrospective review of prospectively managed database	n = 98 104 limbs, 28 CFA stents	Self-expandable (15) and balloon-expandable (13)^a^	ABI from 0.28 to 0.54 (*P* < .001) in CLI and from 0.61 to 0.85 (*P* < .001) in claudicants	1 iatrogenic CFA dissection
						- TLR of 46% and 73% in CLI and 17% and 28% in claudicants at 12/24 months	
						- TER of 35% and 56% in CLI and 24% and 28% in claudicants at 12/24 months	
Silva et al.	2004	N/A	Retrospective	n = 207 CFA stents	Self-expandable^a^	Procedural success 90%	1 patient required surgical revascularization
						- Clinical success 81%	
						- Overall event-free survival 90% at 11.4 months	

Abbreviations: ABI, anklebrachial index; BASI, bioabsorbable stent implantation; CFE, common femoral endarartectomy; CLI, critical limb ischemia; PP, primary patency; PTA, percutaneous transluminal angioplasty; TER, target extremity revascularization; TLR, target lesion revascularization; CFA, common femoral artery.

^a^type of stent not specified.

**Figure 3.**
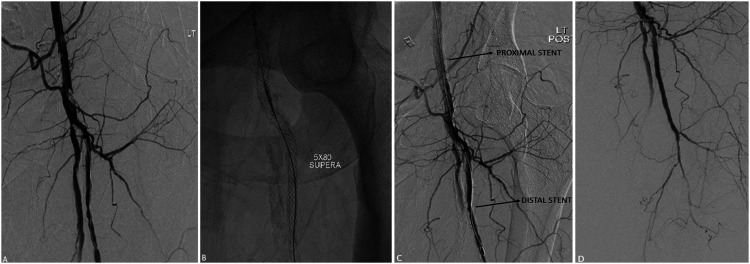
Single stent, jailed superficial femoral artery in a 48-year-old female with history of bilateral lower limb critical limb ischemia, left above-the-knee amputation, and right foot tissue loss. Initial angiogram demonstrates severe left CFA stenosis with proximally patent SFA and multifocal profunda stenosis (A). Left CFA angioplasty to 5 mm and profunda angioplasty to 4 mm. Subsequent deployment of 5 mm x 80 mm SUPERA (B) stent from profunda into the common femoral artery with good angiographic result (C, D).

**Figure 4.**
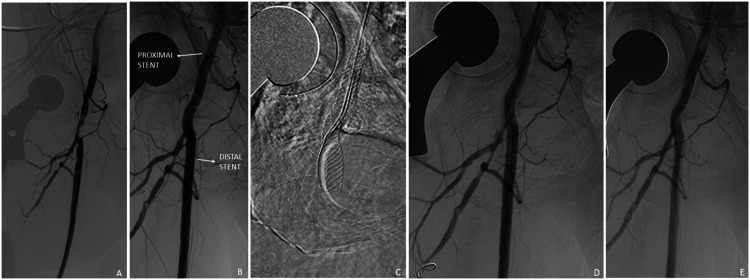
Single stent, jailed profunda femoral artery in a 59-year-old female with acute critical limb ischemia. Initial angiography demonstrated significant stenosis of the common femoral artery and main profunda trunk (A). The common femoral artery was treated with a 6-mm angioplasty balloon with deployment of a 6 x 40 mm SUPERA stent (B). Subsequently, a guidewire was advanced through the SUPERA stent, and the profunda femoral artery was treated with a 3 x 60 mm balloon (C, D) with good angiographic results (E).

**Figure 5.**
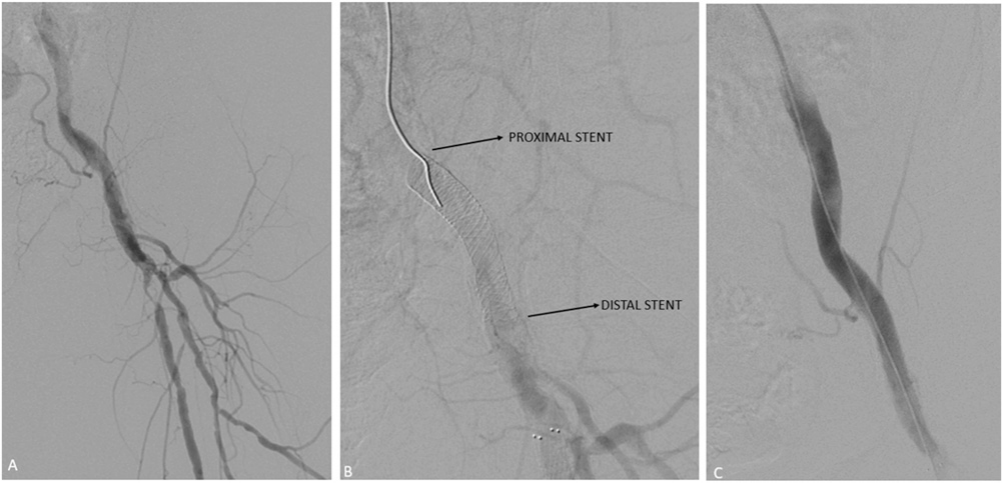
Single stent, isolated common femoral artery. Initial angiogram demonstrated moderate stenosis in the common femoral artery with severe proximal stenosis in the proximal superficial femoral artery (A). The common femoral artery was pre-dilated with an 8-mm angioplasty balloon with subsequent deployment of a 7 x 40 mm SUPERA stent in the common femoral artery (B). Post-angioplasty angiogram revealed adequate angiographic results (C).

**Figure 6.**
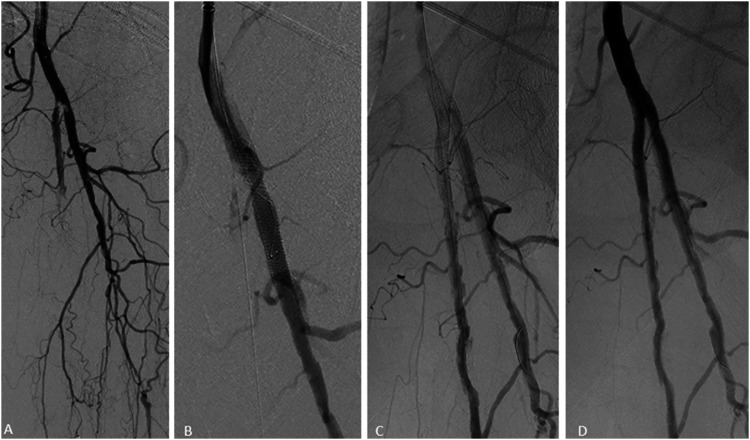
Double kissing stents in a 91-year-old female with critical limb ischemia. Initial angiography demonstrated high-grade stenosis at the origin of the profunda and superficial femoral arteries (A) with deployment of two SUPERA stents (4 x 40 mm and 4 x 40 mm) in a kissing fashion across the bifurcation (B, C) with angiographic improvement (D).

Since the FDA approval of SUPERA stents for peripheral vascular disease in 2014, there has been an upward trend of their usage for selected patients at our institution. Of the 16 patients who had stents implanted, a total of 15 SUPERA stents were placed into the common femoral artery over the study period, all after 2017. In this cohort, technical success was achieved in all cases, and there were no stent-associated complications including fractures or kinking. Complex lesions involving the femoral bifurcation were treated with variety of stenting techniques and can be summarized as single stent, jailed SFA; single stent, jailed profunda femoral artery; single stent, isolated common femoral artery; and double kissing stents. Most cases done at our institution were single stents isolated to the common femoral arteries, while 4 patients had single stented, jailed profunda femoral artery; 2 patients with kissing stents; and 2 with single stented, jailed superficial femoral artery. Examples of each scenario are depicted in Figures 3, 4, 5, and 6.

**Figure 7.**
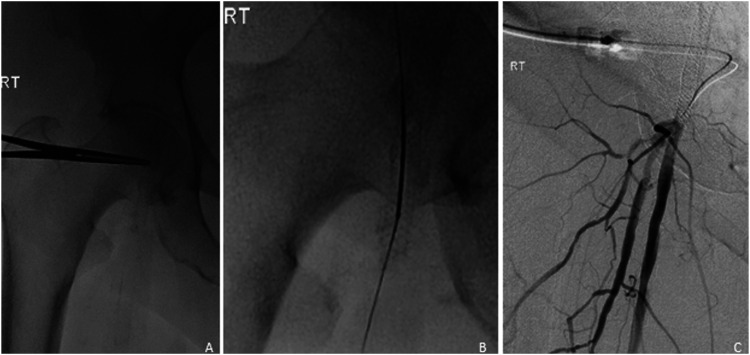
72-year-old female with history of right SUPERA stent placement with repeat angiogram at 13 months for nonhealing ulcer. Under fluoroscopic guidance, the stented right common femoral artery was accessed using a 21-G micropuncture needle. Of note, hemostasis was achieved with a 5-F Exoseal closure device.

**Figure 8.**
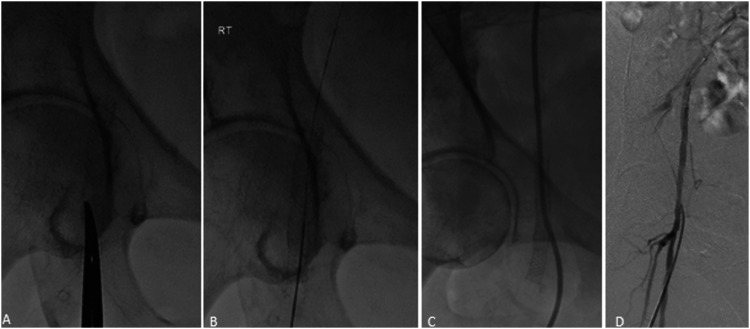
16-month follow-up angiogram. Ultrasound-guided antegrade right CFA access through the SUPERA stent with a 19-G needle (A). Insertion of a 4-Fr dilator over a Bentson wire (B, C). Post-treatment angiogram demonstrated preserved integrity of the stent with no stent-related complications (D).

Implantation of SUPERA stents in the common femoral artery did not preclude or limit future endovascular or surgical revascularization procedures. One patient had multiple re-punctures into the SUPERA stent with placement of vascular sheaths (4-Fr and 6-Fr) and 5-Fr Exoseal vascular closure devices (Cordis Corporation, Bridgewater, New Jersey, USA) during subsequent endovascular interventions with no peri-procedural or subsequent stent compromise (Figure 7). Additionally, the puncturability of the SUPERA stents not only allows for direct vascular accesses but it may also facilitate entry into “jailed” vessels. Figure 4C, D, and E illustrate an example of a SUPERA stent which was deployed from the common femoral artery into the superficial femoral artery. Despite the coverage of the profunda femoral artery ostium, a guidewire successfully traversed the stent and cannulation of the profunda femoral artery was achieved with subsequent angioplasty with satisfactory angiographic results (Figure 8). Additionally, placement of SUPERA stents did not prevent future open surgical revascularization at the common femoral artery as 1 patient in our series underwent a successful secondary endarterectomy for a lesion distal to the patent stented CFA with no complications. Jailing the superficial femoral (n = 1) and profunda femoral arteries (n = 3) with the SUPERA stent did not result in progression or new occlusive lesions in the jailed vessel or compromise potential collateral pathways on follow-up studies.

Given the retrospective nature of this study, a major limitation is its inherent vulnerability to various biases and confounding variables. Additional limitations include its relatively small sample size and the single institutional nature. Despite such limitations, our study particularly highlights the utility and efficacy of SUPERA stents in the CFA and contributes to an area of research which is quite limited in the current literature. Given the significant complexity and heterogeneity in CFA lesions, more robust long-term data are needed to better support this evolving field. At such, endarterectomies remain the mainstay therapeutic option for CFA lesions, whereas endovascular techniques are typically reserved for patients who are poor surgical candidates at our institution. The VMI-CFA (NCT-02804113) and SUPERSURG (NCT-04349657) trials are both ongoing prospective randomized control trials investigating the overall safety and efficacy of endovascular treatment of common femoral artery lesions using SUPERA peripheral stenting systems. These studies will hopefully provide further clarifications on the most appropriate applications and long-term outcomes of endovascular techniques for management of atherosclerotic CFA disease in the future.

## Conclusion

Endovascular revascularization of the common femoral artery is an evolving and highly debated topic in the interventional radiology and vascular surgery community. Despite the recent excitement, there is limited published literature on the overall efficacy of CFA stenting particularly with newer generation stents. Our study provides patient-level data on the outcomes of SUPERA stenting in the common femoral artery.
